# Supplementary material for: Modeling glioblastoma heterogeneity as a dynamic network of cell states
Source: Mol Syst Biol. 2021 Sep 16;17(9):e10105. doi: 10.15252/msb.202010105 (PMC8444284; doi:10.15252/msb.202010105)
Supplement: Supplementary file 6 — Source Data for Figure 5 [file MSB-17-e10105-s004.zip › Figure5A_sourcedata/GSEA_3017/nosigdb_stateC.GseaPreranked.1622795002785/RICHARDS_NOWAKOWSKI_SCIENCE_2017_MGE_IPC1_UPREG.html]

Details for gene set RICHARDS\_NOWAKOWSKI\_SCIENCE\_2017\_MGE\_IPC1\_UPREG[GSEA]

|  || Dataset | state13017 |
| Phenotype | NoPhenotypeAvailable |
| Upregulated in class | na\_pos |
| GeneSet | RICHARDS\_NOWAKOWSKI\_SCIENCE\_2017\_MGE\_IPC1\_UPREG |
| Enrichment Score (ES) | 0.57267106 |
| Normalized Enrichment Score (NES) | 4.395043 |
| Nominal p-value | 0.0 |
| FDR q-value | 0.0 |
| FWER p-Value | 0.0 |
Table: GSEA Results Summary

  

Fig 1: Enrichment plot: RICHARDS\_NOWAKOWSKI\_SCIENCE\_2017\_MGE\_IPC1\_UPREG      
 Profile of the Running ES Score & Positions of GeneSet Members on the Rank Ordered List

  

| PROBE | GENE SYMBOL | GENE\_TITLE | RANK IN GENE LIST | RANK METRIC SCORE | RUNNING ES | CORE ENRICHMENT || 1 | TFDP2 |  |  | 1 | 0.787 | 0.0244 | Yes |
| 2 | HES6 |  |  | 5 | 0.694 | 0.0419 | Yes |
| 3 | ATAD2 |  |  | 7 | 0.649 | 0.0617 | Yes |
| 4 | HIST1H4C |  |  | 9 | 0.603 | 0.0799 | Yes |
| 5 | CENPU |  |  | 10 | 0.588 | 0.0996 | Yes |
| 6 | HIST1H1C |  |  | 11 | 0.583 | 0.1191 | Yes |
| 7 | HIST1H1D |  |  | 12 | 0.583 | 0.1386 | Yes |
| 8 | FAM111A |  |  | 13 | 0.576 | 0.1579 | Yes |
| 9 | EZH2 |  |  | 14 | 0.574 | 0.1771 | Yes |
| 10 | C21orf58 |  |  | 18 | 0.559 | 0.1901 | Yes |
| 11 | RRM2 |  |  | 19 | 0.542 | 0.2082 | Yes |
| 12 | HMGB2 |  |  | 20 | 0.534 | 0.2261 | Yes |
| 13 | CDK6 |  |  | 24 | 0.518 | 0.2376 | Yes |
| 14 | ZBTB20 |  |  | 30 | 0.482 | 0.2442 | Yes |
| 15 | UBE2T |  |  | 31 | 0.475 | 0.2601 | Yes |
| 16 | HIST1H1B |  |  | 35 | 0.460 | 0.2697 | Yes |
| 17 | GMNN |  |  | 38 | 0.453 | 0.2810 | Yes |
| 18 | ORC6 |  |  | 39 | 0.450 | 0.2961 | Yes |
| 19 | ESCO2 |  |  | 43 | 0.440 | 0.3051 | Yes |
| 20 | UHRF1 |  |  | 45 | 0.436 | 0.3177 | Yes |
| 21 | RAD51AP1 |  |  | 46 | 0.435 | 0.3323 | Yes |
| 22 | TMPO |  |  | 49 | 0.432 | 0.3429 | Yes |
| 23 | HIST1H1E |  |  | 51 | 0.430 | 0.3554 | Yes |
| 24 | TOP2A |  |  | 52 | 0.426 | 0.3696 | Yes |
| 25 | SPC25 |  |  | 53 | 0.415 | 0.3835 | Yes |
| 26 | TYMS |  |  | 60 | 0.390 | 0.3851 | Yes |
| 27 | SMC2 |  |  | 63 | 0.386 | 0.3941 | Yes |
| 28 | RAB3IP |  |  | 66 | 0.382 | 0.4031 | Yes |
| 29 | GLCCI1 |  |  | 68 | 0.378 | 0.4138 | Yes |
| 30 | DEK |  |  | 74 | 0.373 | 0.4167 | Yes |
| 31 | SMC4 |  |  | 78 | 0.365 | 0.4232 | Yes |
| 32 | ITGB3BP |  |  | 79 | 0.364 | 0.4353 | Yes |
| 33 | CDCA5 |  |  | 81 | 0.361 | 0.4455 | Yes |
| 34 | BARD1 |  |  | 86 | 0.357 | 0.4498 | Yes |
| 35 | MIS18BP1 |  |  | 89 | 0.355 | 0.4578 | Yes |
| 36 | USP1 |  |  | 92 | 0.352 | 0.4657 | Yes |
| 37 | ASRGL1 |  |  | 93 | 0.350 | 0.4774 | Yes |
| 38 | NUSAP1 |  |  | 100 | 0.340 | 0.4773 | Yes |
| 39 | CKB |  |  | 102 | 0.335 | 0.4866 | Yes |
| 40 | ZNF43 |  |  | 107 | 0.330 | 0.4899 | Yes |
| 41 | H2AFX |  |  | 115 | 0.322 | 0.4873 | Yes |
| 42 | CCDC34 |  |  | 120 | 0.318 | 0.4902 | Yes |
| 43 | FRMD4B |  |  | 123 | 0.313 | 0.4969 | Yes |
| 44 | RHOBTB3 |  |  | 124 | 0.313 | 0.5074 | Yes |
| 45 | HMGN2 |  |  | 126 | 0.312 | 0.5159 | Yes |
| 46 | FBXO5 |  |  | 130 | 0.308 | 0.5204 | Yes |
| 47 | H3F3A |  |  | 132 | 0.304 | 0.5287 | Yes |
| 48 | KIF15 |  |  | 136 | 0.302 | 0.5330 | Yes |
| 49 | NCAPG |  |  | 137 | 0.301 | 0.5431 | Yes |
| 50 | CENPK |  |  | 139 | 0.299 | 0.5512 | Yes |
| 51 | LMNB1 |  |  | 142 | 0.293 | 0.5571 | Yes |
| 52 | ASCL1 |  |  | 145 | 0.290 | 0.5630 | Yes |
| 53 | C12orf57 |  |  | 146 | 0.289 | 0.5727 | Yes |
| 54 | PHF6 |  |  | 155 | 0.285 | 0.5668 | No |
| 55 | CHD7 |  |  | 166 | 0.277 | 0.5569 | No |
| 56 | VRK1 |  |  | 169 | 0.275 | 0.5623 | No |
| 57 | MAD2L1 |  |  | 174 | 0.270 | 0.5636 | No |
| 58 | NCAPG2 |  |  | 187 | 0.260 | 0.5493 | No |
| 59 | CDK1 |  |  | 191 | 0.255 | 0.5521 | No |
| 60 | TNFAIP8L1 |  |  | 215 | -0.257 | 0.5165 | No |
| 61 | ARL4A |  |  | 224 | -0.259 | 0.5098 | No |
| 62 | MBNL2 |  |  | 233 | -0.260 | 0.5032 | No |
| 63 | CLIC1 |  |  | 237 | -0.261 | 0.5062 | No |
| 64 | RPL12 |  |  | 332 | -0.288 | 0.3354 | No |
| 65 | RPL22L1 |  |  | 404 | -0.322 | 0.2099 | No |
| 66 | HMGCR |  |  | 418 | -0.334 | 0.1961 | No |
| 67 | LDLR |  |  | 419 | -0.334 | 0.2073 | No |
| 68 | WWTR1 |  |  | 437 | -0.345 | 0.1862 | No |
| 69 | FAT1 |  |  | 441 | -0.346 | 0.1920 | No |
| 70 | HSPA5 |  |  | 447 | -0.350 | 0.1942 | No |
| 71 | B2M |  |  | 468 | -0.371 | 0.1682 | No |
| 72 | PTPRE |  |  | 469 | -0.372 | 0.1806 | No |
| 73 | RAB13 |  |  | 482 | -0.388 | 0.1706 | No |
| 74 | LDHA |  |  | 498 | -0.412 | 0.1556 | No |
| 75 | TGIF1 |  |  | 502 | -0.421 | 0.1639 | No |
| 76 | NES |  |  | 509 | -0.435 | 0.1670 | No |
Table: GSEA details [plain text format]

  

Fig 2: RICHARDS\_NOWAKOWSKI\_SCIENCE\_2017\_MGE\_IPC1\_UPREG: Random ES distribution      
 Gene set null distribution of ES for **RICHARDS\_NOWAKOWSKI\_SCIENCE\_2017\_MGE\_IPC1\_UPREG**
